# A dominant negative mutation of transforming growth factor- **β** receptor type II gene in microsatellite stable oesophageal carcinoma

**DOI:** 10.1054/bjoc.1999.1178

**Published:** 2000-04-03

**Authors:** Shinji Tanaka, Masaki Mori, Ken–ichi Mafune, Shinji Ohno, Keizo Sugimachi

**Affiliations:** Department of Surgery II, Faculty of Medicine, Department of Surgery, Medical Institute of Bioregulation, Kyushu University, 3-1-1 Maidashi, Fukuoka, 812-8582, Japan; Department of Surgery, Faculty of Medicine, University of Tokyo, Japan

**Keywords:** TGF-β RII, Smad2, Smad4, oesophageal carcinoma, microsatellite

## Abstract

Recent investigations revealed microsatellite instability in colon cancers are associated with mutations of the transforming growth factor-β receptor type II gene (TGF-β RII) that encodes a transmembrane protein containing an intracellular serine/threonine kinase domain. Activation of TGF-β receptor type I (RI) and RII by TGF-β induces nuclear translocation of Smad proteins including Smad2 and Smad4 that have been originally identified as tumour suppressor genes. We have previously reported six cases with microsatellite instability in 32 oesophageal carcinomas. In this study, we analysed genetic mutations of TGF-β RII, Smad2 and Smad4 in these oesophageal carcinoma tissues and established 16 cell lines. No genetic mutation was detected in any tissues or cell lines except one tissue sample of microsatellite stable oesophageal carcinoma, that is, a mis-sense mutation of glutamic acid to glutamine at codon 526 (E526Q) in the TGF-β RII serine/threonine kinase domain. Interestingly, the mutant TGF-β RII E526Q can completely inhibit TGF-β-induction of nuclear translocation of Smad4 protein in oesophageal carcinoma cells. This mutation of TGF-β RII that is not associated with microsatellite instability might make a dominant negative effect on TGF-β signal transduction in oesophageal carcinoma. © 2000 Cancer Research Campaign


British Journal of Cancer (2000) 82(9), 1557–1560
© 2000 Cancer Research Campaign
DOI: 10.1054/ bjoc.1999.1178, available online at http://www.idealibrary.com on IDEAL®

### A dominant negative mutation of transforming growth factor-β receptor type II gene in microsatellite stable oesophageal carcinoma

**Shinji Tanaka[1], Masaki Mori[2], Ken–ichi Mafune[3], Shinji Ohno[1] and Keizo Sugimachi[1]**

[1]Department of Surgery II, Faculty of Medicine and [2]Department of Surgery, Medical Institute of Bioregulation, Kyushu University, 3-1-1 Maidashi, Fukuoka 812-8582, Japan; [3]Department of Surgery, Faculty of Medicine, University of Tokyo, Japan

**Summary** Recent investigations revealed microsatellite instability in colon cancers are associated with mutations of the transforming growth factor-β receptor type II gene (TGF-β RII) that encodes a transmembrane protein containing an intracellular serine/threonine kinase domain. Activation of TGF-β receptor type I (RI) and RII by TGF-β induces nuclear translocation of Smad proteins including Smad2 and Smad4 that have been originally identified as tumour suppressor genes. We have previously reported six cases with microsatellite instability in 32 oesophageal carcinomas. In this study, we analysed genetic mutations of TGF-β RII, Smad2 and Smad4 in these oesophageal carcinoma tissues and established 16 cell lines. No genetic mutation was detected in any tissues or cell lines except one tissue sample of microsatellite stable oesophageal carcinoma, that is, a mis-sense mutation of glutamic acid to glutamine at codon 526 (E526Q) in the TGF-β RII serine/threonine kinase domain. Interestingly, the mutant TGF-β RII E526Q can completely inhibit TGF-β-induction of nuclear translocation of Smad4 protein in oesophageal carcinoma cells. This mutation of TGF-β RII that is not associated with microsatellite instability might make a dominant negative effect on TGF-β signal transduction in oesophageal carcinoma. © 2000 Cancer Research Campaign

**Keywords**: TGF-β RII; Smad2; Smad4; oesophageal carcinoma; microsatellite

Transforming growth factor β (TGF-β) is a pre-eminent negative growth factor and/or an apoptotic inducer for epithelial cells (Hata et al, 1998). Work over the past few years has led to the elucidation of a TGF-β signal transduction network (Massague, 1998). TGF-β induces diverse cellular changes through heteromeric complexes of TGF-β receptor type I (TGF-β RI) and receptor type II (TGF-β RII), such that the loss of either receptor dramatically alters the cellular response (Wrana et al, 1994). This network involves the receptor serine/threonine kinases at the cell surface and their substrates, Smad proteins (mothers against decapentaplegic), which move into the nucleus, where they activate target gene transcription in association with DNA-binding partners (Kretzschmar and Massague 1998).

Mutations in these pathways of TGF-β signalling are the cause of various forms of human malignancies (Hata et al, 1998). Among the Smad family members, Smad2 and Smad4 have been originally isolated as tumour suppressor gene MADR2 and DPC4 respectively (Eppert et al, 1996; Hahn et al, 1996). An additional role for TGF-β RII as a tumour suppressor gene was demonstrated by the discovery of inactivating the genome mutations in the tandem repeat segments, namely microsatellites due to replication error in colorectal cancers (Markowitz et al, 1995; Wang et al, 1995). The A10 microsatellite, which is within the coding region of the extracellular domain of the Type II receptor, is prone to 1 bp deletions that introduce a stop codon, leading to the protein truncation, and resistance to TGF-β's antiproliferative effect (Grady et al, 1999). Another unstable microsatellite region has been documented in colon carcinoma: two copies of a dinucleotide repeat (GTGTGT) that, when expanded, lead to an altered C-terminal amino acid sequence for the receptor (Grady et al, 1999). In this regard, the colorectal microsatellite instability might play an important role in the pathogenesis. More recently, frequent mutations of TGF-β RII gene are identified also in colorectal cancers without microsatellite instability (Grady et al, 1999), suggesting mutational down-regulation of TGF-β signalling might be targeted not only by the replication error phenotype.

We have previously reported less frequency of microsatellite instability in oesophageal carcinomas (Nakashima et al, 1995), one of the aggressive human cancers (Sugimachi et al, 1994). In this study, we analysed genetic mutations of TGF-β RII, Smad2, and Smad4 in these oesophageal samples with microsatellite instability or stability. Additionally, an effect of our detected mutation on the TGF-β signalling was analysed in cultured oesophageal carcinoma cells. This is the first report regarding the relationship between microsatellite instability and mutational inactivation of TGF-β in human oesophageal carcinoma.

### MATERIALS AND METHODS

We have previously reported detection of microsatellite instability in six cases of 32 human oesophageal carcinomas obtained from surgical resection (Nakashima et al, 1995). The genetic mutation of TGF-β RII, Smad2 and Smad4 was studied in these carcinomas with or without microsatellite instability and adjacent normal mucosa. The extracted DNA from tissue samples were used for further PCR analysis as described previously (Tanaka et al, 1993)

*Received 22 July 1999*
*Revised 22 November 1999*
*Accepted 24 November 1999*

*Correspondence to:* S Tanaka using the following primers; *TGF-β RII* 5′-TCGGTCTATGACGAGCAG-3′, 5′-GGGACCCCAGGAAGACC-3′ (exon 1), 5′-GGGCTGGTATCAAGTTCATTTG-3′, 5′-GGAGACAGAGATACACTGACTGTG-3′ (exon 2), 5′-TCCAATGAATCTCTTCACTC-3′, 5′-CCCACACCCTTAAGAGAAGA-3′ (exon 3), 5′-CCAACTCCTTCTCTCCTTGTTTTG-3′, 5′-TCCAAGAGGCATACTCCTCATAGG-3′ (exon 4), 5′-GTCGCTTTGCTGAGGTCTATAAGG-3′, 5′-CCAGGCTCAAGGTAAAGGGGATCTAGCA-3′ (exon 4), 5′-GGCGCTGGAATTAAATGATGGGC-3′, 5′-GAATAATGCTCGAAGCAACACATG-3′ (exon 5), 5′-TTTCCTTTGGGCTGCACATG-3′, 5′-CCTAAGAGGCAACTTGGTTGAATC-3′ (exon 6), 5′-CCAACTCATGGTGTCCCTTTG-3′, 5′-TCTTTGGACATGCCCAGCCTG-3′ (exon 7); *Smad 2* 5′-AATCAGCGGGCGGCAGGGT-3′, 5′-AGCCCACCTCCGCTTCCG-3′ (exon 1), 5′-AAGACGGCGGCCGGGAGT-3′, 5′-ACGCAAACACTTCCCTAGCT-3′ (exon 1), 5′-GGTAGTCTCTACATCATCCT-3′, 5′-GGCAACTTGAAAGGAACACA-3′ (exon 2), 5′-AGTAACCAGCACTACATGCCTGTG-3′, 5′-CTTTCAAAATATACCCCCCTCCC-3′ (exon 3), 5′-ATCTGCTAGTGCTGCTGCA-3′, 5′-CCTGGGTCACAAGAGTACT-3′ (exon 4), 5′-GTAGGTGGACCCTAGCTTT-3′, 5′-TTAGGAGATTCAGAAGGCAA-3′ (exon 5), 5′-GGTAGCTGAGAGAAAAGGTAGTG-3′, 5′-TTGGTATGCGTCTCAACTTC-3′ (exon 6), 5′-GCCAAAACTGTTGCACCTT-3′, 5′-GTGCCAGCAGAAAAGACTT-3′ (exon 7), 5′-ACTTCCTGAGCTTTTGCCAGTG-3′, 5′-TGCACACAAGCTCTTGATGTGG-3′ (exon 8), 5′-GCTTCCAAAGTCACACTGA-3′, 5′-ATTTGGAGGCCTCCAACTT-3′ (exons 9–10), 5′-GCATGTACCTAAACATAGC-3′, 5′-CAGAGAAGTGGGAATAACAG-3′ (exon 11); *Smad4* 5′-CGTTAGCTGTTGTTTTCACTG-3′, 5′-AGAGTATGTGAAGAGATGGAG-3′ (exon 1), 5′-TGTATGACATGGCCAAGTTAG-3′, 5′-CAATACTCGGTTTTAGCAGTC-3′ (exon 2), 5′-CTGAATTGAAATGGTTCATGAAC-3′, 5′-GCCCCTAACCTCAAAATCTAC-3′ (exon 3), 5′-TTTTGCTGGTAAAGTAGTATGC-3′, 5′-CTATGAAAGATAGTACAGTTAC-3′ (exon 4), 5′-CATCTTTATAGTTGTGCATTATC-3′, 5′-TAATGAAACAAAATCACAGGATG-3′ (exons 5–6), 5′-TGAAAGTTTTAGCATTAGACAAC-3′, 5′-TGTACTCATCTGAGAAGTGAC-3′ (exon 7), 5′-TGTTTTGGGTGCATTACATTTC-3′, 5′-CAATTTTTTAAAGTAACTATCTGA-3′ (exon 8), 5′-TATTAAGCATGCTATACAATCTG-3′, 5′-CTTCCACCCAGATTTCAATTC-3′ (exon 9), 5′-AGGCATTGGTTTTTAATGTATG-3′, 5′-CTGCTCAAAGAAACTAATCAAC-3′ (exon 10), 5′-CCAAAAGTGTGCAGCTTGTTG-3′, 5′-CAGTTTCTGTCTGCTAGGAG-3′ (exon) 11). After screening by polymerare chain reaction-single strand conformation polymorphisms (PCR-SSCP) method (Tanaka et al, 1993), the PCR products were directly sequenced by a cycle sequencing procedure (Applied Biochemical Inc.). Sixteen of cultured cell lines of human oesophageal carcinoma cells were also analysed (KSE-1, KSE-2, KYSE-30, KYSE-50, KYSE-110, KYSE-150, KYSE-180, KYSE-200, KYSE-220, KYSE-410, KYSE-450, TE-1, TE-2, TE-3, TE-4 and TE-5).

The effect of mutant TGF-β RII was assessed by transient transfection on a human oesophageal carcinoma cell line using Lipofectamine (Gibco-BRL, Life Technologies, Inc., Gaithersburg, MD, USA) (Tanaka et al, 1998). The expression plasmids of TGF-β RII and Smad4 were kindly provided from Dr Michael Reiss (Yale University) and Dr Joan Massague (Memorial Sloan-Kettering Cancer Center) respectively. The mutant TGF-β RII lacking the serine/threonine kinase domain (ΔKD) was generated by introduction of a stop codon just before the kinase domain using the PCR method (Tanaka et al, 1998). Following 10 ng $ml^{-1}$ of TGF-β1 stimulation, the transfected FLAG-tagged protein was detected by indirect immunofluorescent technique using anti-FLAG M2 mouse monoclonal antibody (Eastman Kodak Co., Rochester, NY, USA) and anti-immunoglobulin preparation conjugated with fluorescence (CAPPEL Research Products, Durham, NC, USA), as described previously (Tanaka et al, 1998). The results of cell count were determined by an independent pathologist from three separate experiments.

### RESULTS

First, we performed PCR-SSCP and sequencing analysis to detect genetic mutations of TGF-β RII gene, Smad2 and Smad4 in 32 oesophageal carcinoma tissues, adjacent normal mucosa and 16 carcinoma cell lines (Table 1). Of these 32 carcinomas, microsatellite instability was identified in six cases (Nakashima et al, 1995). We cannot detect any genetic mutation in Smad2 and Smad4, that are frequently mutated in colon and pancreas carcinomas respectively (Eppert et al, 1996; Hahn et al, 1996). On the other hand, a mis-sense mutation of TGF-β RII gene was detected in one tissue sample of oesophageal carcinoma showing microsatellite stability (Nakashima et al, 1995). This missense mutation of TGF-β RII gene (GAC→CAC) caused amino acid substitution from glutamic acid to glutamine at residue 526 (E526Q). As shown in Figure 1, the wild-type allele of the gene was also recognized together. Interestingly, the same mutation has been reported in a TGF-β-resistant cell line of head and neck carcinoma (Garrigue-Antar et al, 1995). The amino acid of residue 526 in the TGF-β RII protein, located within intracellular serine/threonine kinase domain (Figure 2A), was revealed to be essential for the kinase activity (De et al, 1998).

Then, we investigated the effects of the mutant E526Q expression in the oesophageal carcinoma cells. A human oesophageal carcinoma cell line KYSE150 was revealed to be sensitive to TGF-β signals without the mutant components such as RII, Smad2 and Smad4. The mammalian expression vectors of mutant E526Q were co-transfected together with Smad4 expression vector tagged with the FLAG-epitope into the KYSE150 oesophageal carcinoma cell line, followed by analysis of location of the Smad4 protein. As shown in Figure 2B, nuclear accumulation of Smad4 protein was recognized in the transfected KYSE150 cells under TGF-β stimulation, indicating intracellular transduction of TGF-β signals. On the other hand, ectopic expression of the E526Q inhibited the TGF-β-induced nuclear translocation of Smad4 protein that was detected in cytoplasm. The similar inhibitory effect was also observed by transfection of TGF-β RII mutant lacking the kinase domain (ΔKD), that is known as the typical dominant negative mutant (Wrana et al, 1994). Therefore, the mutation of TGF-β RII

**Table 1** Genetic mutation of TGF-β RII, Smad2 and Smad4 in human oesophageal carcinoma tissues and cell lines

| Samples | Total | TGF-β RII | Smad2 | Smad4 |
|---|---|---|---|---|
| Carcinoma tissues | | | | |
| Unstable | 6 | 0 | 0 | 0 |
| Stable | 26 | 1 (E526Q/wt) | 0 | 0 |
| Normal mucosa tissues | 32 | 0 | 0 | 0 |
| Cell lines | 16 | 0 | 0 | 0 |

*© 2000 Cancer Research Campaign*

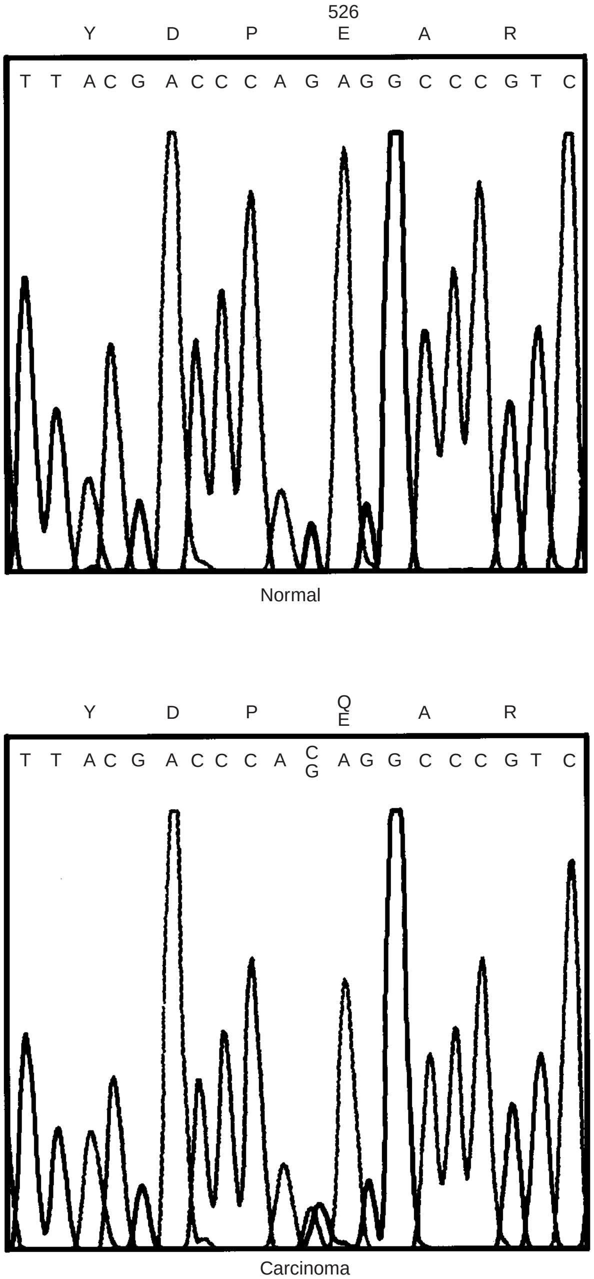

**Figure 1** Sequence analysis of TGF-β RII gene from oesophageal tissue of normal mucosa (upper), and carcinoma (lower). While nucleotides GAG encoding glutamic acid (E) at residue 526 was in normal mucosa, the nucleotides C̲AG encoding glutamine (Q) were also found with GAG (E) in oesophageal carcinoma

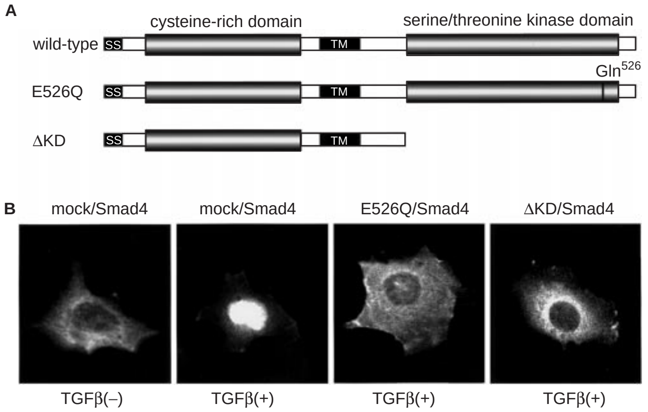

**Figure 2** Effects of the mutant receptor type II on TGF-β signal transduction for Smad4 protein. (**A**) Schematic representation demonstrating the structural and functional domains of wild-type TGF-β RII protein, the missense mutant E526Q, and the kinase domain-defect mutant ΔKD. SS, signal sequence; TM, transmembrane region. (**B**) Localization of FLAG-tagged Smad4 protein in co-transfected KYSE150 cells. Nuclear accumulation of Smad4 protein in 40.7 ± 3.5% of mock plasmid cotransfectants after TGF-β stimulation, but only in 2.1 ± 0.5% of unstimulated mock cotransfectants, as shown in the panels of mock/Smad4/TGF-β(+) and mock/Smad4/TGF-β(–). Such TGF-β-induced translocation was abolished in contransfectantion with the missense mutant TGF-β RII E526Q (3.2 ± 0.9%) or the carboxyl truncated mutant ΔKD (3.9 ± 0.8%), as shown in the right two panels. FLAG-tagged Smad4 was detected by anti-FLAG antibody M2 followed by immunofluorescent secondary antibody, and the results were determined from three independent experiments recognized in microsatellite stable oesophageal carcinoma might make a dominant negative effect on TGF-β signal transduction in oesophageal carcinoma cells.

### DISCUSSION

The microsatellite instability can result from a replication error-prone phenotype associated with mutations in human DNA mismatch repair genes homologous to bacterial genes *MutS* and *MutL*, such as *hMSH2*, *hMSH3*, *hMSH6*/*GTBP*, *hMLH1*, *hPMS1* and *hPMS2* (Fishel, 1998). The discovery of a mutation in one of the principle components of the TGF-β receptor system which is linked to a DNA repair defect (Markowitz et al, 1995; Wang et al, 1995) represents one possible mechanism of escape from negative regulatory influences acting upon cells. While microsatellite instability is identified in almost all malignancies, the frequency is variously ranged among carcinoma types. Oesophageal carcinoma has less frequency of instability as shown in our previous reports (Nakashima et al, 1995). This study demonstrated no genetic mutation of TGF-β RII gene in the oesophageal carcinomas with microsatellite instability. It is noteworthy that one mutant TGF-β RII was found in a sample of microsatellite stable oesophageal carcinoma. This mis-sense mutation substituted a glutamic acid to glutamine at residue 526 (E526Q) in the strictly conserved region of serine/threonine kinase domain (De et al, 1998). The same mutation has been detected in a neck and head carcinoma cell line, SqCC/Y1 (Garrigue-Antar et al, 1995). Interestingly, two microsatellite stable colorectal cancers were recently reported to have mutant TGF-β RII at residue 522 and residue 528 (Grady et al, 1999). These mutations are located within kinase subdomain XI, that is essential for the serine/threonine kinase activity. Thus, this region XI might be the mutational hot-spots of TGF-β RII gene independently of microsatellite instability.

TGF-β, one of the potentently negative regulators of epithelial cells (Hata et al, 1998), induces intracellular signals through heteromeric receptor complexes of TGF-β RI and TGF-β RII serine/threonine receptors (Massague, 1998). Receptor activation occurs upon binding of ligand to TGF-β RII, which then recruits

© 2000 Cancer Research Campaign 

and phosphorylates TGF-β RI, which propagates the signal to downstream targets, Smad family proteins. Human Smad family is composed of at least eight different proteins Smad1–8 that are classified into three types (Wrana et al, 1994). Upon stimulating pathway, Class I Smads (Smad1/5/8 and Smad2/3) have been shown to be directly phosphorylated by TGF-β RI serine/threonine kinase. Class II Smads (Smad4), referred to as 'co-Smads', forms a complex with Class I Smads, which take it into the nucleus. Formation of this complex is required for optimal binding and transcriptional activation of target genes in TGF-β responses. Class III Smads (Smad 6/7) block phosphorylation of the Class I Smads by binding to the cytoplasmic portion of the receptors. Expression of the 'antagonistic Smads' is induced in response to the ligands, suggesting that antagonistic Smads might participate in negative feedback loops to regulate the intensity and duration of TGF-β responses. Smad2 of Class I and Smad4 of Class II have been also identified as tumour suppressor genes mutated in colorectal cancers and pancreatic cancers respectively. In our present study, any genetic mutation of Smad2 and Smad4 is not observed in either microsatellite unstable or stable cancers of oesophagus, suggesting that no Smad might function as tumour suppressor genes in oesophageal carcinogenesis.

There have been several mechanisms reported to acquire resistance to TGF-β in cancer cells. For example, we have previously identified anti-apoptotic signal transduction against TGF-β, although intracellular TGF-β signals is clearly recognized in hepatocellular carcinoma cells (Tanaka and Wands, 1996). By extension, it seems likely that other antiproliferative or apoptotic genes could acquire mutations, thereby conferring resistance and monoclonal expansion. It is also important to consider the possibility that nonmutagenic events could confer resistance to TGF-β's antiproliferative effects. Since mutational inactivation of TGF-β signalling pathways is not frequently occurred under microsatellite unstable conditions in oesophageal carcinoma, functional down-regulation or other genetic instability of TGF-β signalling network should be further investigated (Lengauer et al, 1998).

### ACKNOWLEDGEMENTS

We thank M Reiss for providing K526Q cDNA and J. Massague for providing Smad4 cDNA. We also thank K Taguchi for immunocytochemical analysis; K Ogata and T Shimooka for technical assistance. This work was supported by Fukuoka Cancer Society, Sagawa Research Fundation, and a Grant-in-Aid from Ministry of Education, Science, Sports and Culture of Japan.

### REFERENCES

De M, Yan W, de Jonge RR, Garrigue-Antar L, Vellucci VF and Reiss M (1998) Functional characterization of transforming growth factor β type II receptor mutants in human cancer. *Cancer Res* **58**: 1986–1992

Eppert K, Scherer SW, Ozcelik H, Pirone R, Hoodless P, Kim H, Tsui LC, Bapat B, Gallinger S, Andrulis IL, Thomsen GH, Wrana JL and Attisano L (1996) MADR2 maps to 18q21 and encodes a TGFβ-regulated MAD-related protein that is functionally mutated in colorectal carcinoma. *Cell* **86**: 543–552

Fishel R (1998) Mismatch repair molecular switches and signal transduction. *Genes Dev* **12**: 2096–2101

Garrigue-Antar L, Munoz-Antonia T, Antonia SJ, Gesmonde J, Vellucci VF and Reiss M (1995) Missense mutations of the transforming growth factor β type II receptor in human head and neck squamous carcinoma cells. *Cancer Res* **55**: 3982–3987

Grady WM, Myeroff LL, Swinler SE, Rajput A, Thiagalingam S, Lutterbaugh JD, Neumann A, Brattain MG, Chang J, Kim SJ, Kinzler KW, Vogelstein B, Willson JK and Markowitz S (1999) Mutational inactivation of transforming growth factor β receptor type II in microsatellite stable colon cancers. *Cancer Res* **59**: 320–324

Hata A, Shi Y and Massague J (1998) TGF-β signaling and cancer: structural and functional consequences of mutations in Smads. *Mol Med Today* **4**: 257–262

Hahn SA, Schutte M, Hoque AT, Moskaluk CA, da Costa LT, Rozenblum E, Weinstein CL, Fischer A, Yeo CJ, Hruban RH and Kern SE (1996) DPC4 a candidate tumor suppressor gene at human chromosome 18q21.1. *Science* **271**: 350–353

Kretzschmar M and Massague J (1998) SMADs: mediators and regulators of TGF-β signaling. *Curr Opin Genet Dev* **8**: 103–111

Lengauer C, Kinzler KW and Vogelstein B (1998) Genetic instabilities in human cancers. *Nature* **396**: 643–649

Markowitz S, Wang J, Myeroff L, Parsons R, Sun LZ. Lutterbaugh J, Fan RS, Zborowska E, Kinzler KW, Vogelstein B, et al (1995) Inactivation of the type II TGF-β receptor in colon cancer cells with microsatellite instability. *Science* **268**: 1336–1338

Massague J (1998) TGF-β signal transduction. *Annu Rev Biochem* **67**: 753–791

Nakashima H, Mori M, Mimori K, Inoue H, Shibuta K, Baba K, Mafune K and Akiyoshi T (1995) Microsatellite instability in Japanese esophageal carcinoma. *Int J Cancer* **64**: 286–289

Sugimachi K, Watanabe M, Sadanaga N, Ikebe M, Kitamura K, Mori M and Kuwano H (1994) Recent advances in the diagnosis and surgical treatment of patients with carcinoma of the esophagus. *J Am Coll Surg* **178**: 363–368

Tanaka S and Wands JR (1996) Insulin receptor substrate 1 overexpression in human hepatocellular carcinoma cells prevents transforming growth factor β-induced apoptosis. *Cancer Res* **56**: 3391–3394

Tanaka S, Toh Y, Adachi E, Matsumata T, Mori R and Sugimachi K (1993) Tumor progression in hepatocellular carcinoma may be mediated by p53 mutation. *Cancer Res* **53**: 2884–2887

Tanaka S, Akiyoshi T, Mori M, Wands JR and Sugimachi K (1998) A novel frizzled gene identified in human esophageal carcinoma that mediates APC/β-catenin signals. *Proc Natl Acad Sci USA* **95**: 10164–10169

Wang J, Sun LZ, Myeroff L, Wang XF, Gentry LE, Yang J, Liang J, Zborowska E, Markowitz S, Willson JKV and Brattain MG (1995) Demonstration that mutation of the type II transforming growth factor receptor β inactivates its tumor suppressor activity in replication error-positive colon carcinoma cells. *J Biol Chem* **270**: 22044–22049

Wrana JL, Attisano L, Wieser R, Ventura F and Massague J (1994) Mechanism of activation of the TGF-β receptor. *Nature* **370**: 341–347

 *© 2000 Cancer Research Campaign*
